# Cathepsin H deficiency decreases hypoxia-ischemia-induced hippocampal atrophy in neonatal mice through attenuated TLR3/IFN-β signaling

**DOI:** 10.1186/s12974-021-02227-7

**Published:** 2021-08-10

**Authors:** Junjun Ni, Juan Zhao, Xinwen Zhang, Thomas Reinheckel, Vito Turk, Hiroshi Nakanishi

**Affiliations:** 1 Key Laboratory of Molecular Medicine and Biotherapy, School of Life Science, Beijing Institute of Technology, Beijing, 100081 China; 2 Department of Aging Science and Pharmacology, Faculty of Dental Science, Kyushu University, Fukuoka, 812-8582 Japan; 3 Center of Implant Dentistry, School of Somatology, China Medical University, Shenyang, 110122 China; 4 Institute of Molecular Medicine and Cell Research, University of Freiburg, 79104 Freiburg, Germany; 5 Department of Biochemistry and Molecular and Structural Biology, J. Stefan Institute, Ljubljana, Slovenia; 6 Department of Pharmacology, Faculty of Pharmacy, Yasuda Women’s University, Hiroshima, 731-0153 Japan

**Keywords:** Cathepsin H, Interferon-β, Microglia/Macrophages, Astrocyte

## Abstract

**Background:**

Cathepsin H (CatH) is a lysosomal cysteine protease with a unique aminopeptidase activity. Its expression level is increased in activated immune cells including dendritic cells, macrophages, and microglia. We have previously reported that CatH deficiency impairs toll-like receptor 3 (TLR3)-mediated activation of interferon regulatory factor 3 (IRF3), and the subsequent secretion of interferon (IFN)-β from dendritic cells. Furthermore, there is increasing evidence that IFN-β secreted from microglia/macrophages has neuroprotective effects. These observations prompted further investigation into the effects of CatH deficiency on neuropathological changes.

**Methods:**

In this study, neuropathological changes were examined using histochemical staining (both hematoxylin-eosin (H&E) and Nissl) of the hippocampus of wild-type (WT) and CatH-deficient (*CatH*^−/−^) mice after hypoxia-ischemia (HI). The density and the localization of CatH and TLR3 were examined by immunofluorescent staining. CatH processing in microglia was assayed by pulse-chase experiments, while immunoblotting was used to examine TLR3 expression and IRF3 activation in microglia/macrophages in the presence of poly(I:C). Microglial cell death was examined by fluorescence-activated cell sorting (FACS), and primary astrocyte proliferation in the presence of IFN-β was examined using scratch wound assay.

**Results:**

WT mice displayed severe atrophy in association with neuronal death and moderate astrogliosis in the hippocampus following neonatal HI. Somewhat surprisingly, *CatH*^−/−^ mice showed marked neuronal death without severe atrophy in the hippocampus following HI. Furthermore, there was notable microglia/macrophages cell death and strong astrogliosis in the hippocampus. The TLR3 and phosphorylated IRF3 expression level in the hippocampus or splenocytes (mainly splenic macrophages); from *CatH*^−/−^ mice was lower than in WT mice. In vitro experiments demonstrated that recombinant IFN-β suppressed HI-induced microglial cell death and astrocyte proliferation.

**Conclusion:**

These observations suggest that CatH plays a critical role in the proteolytic maturation and stabilization of TLR3, which is necessary for IFN-β production. Therefore, impaired TLR3/IFN-β signaling resulting from CatH deficiency may induce microglial cell death after activation and astrogliosis/glial scar formation in the hippocampus following HI injury, leading to suppression of hippocampal atrophy.

## Background

Neonatal hypoxia-ischemia (HI) induces brain injury, causing neurological impairment, including cognitive impairment, motor dysfunction, and seizures [1]. Glial cells play a crucial role in the pathomechanism of neonatal HI [2, 3]. Microglia, the resident mononuclear phagocyte population in the brain, play essential roles in chronic neuroinflammation, which constitutes a fundamental process involved in the progression of neuronal death after HI injury. Microglia are the first cell type to respond to early neuronal death caused by HI. Activated microglia play a central role in triggering neuroinflammation, leading to delayed cell death of other neurons and subsequent immature cerebral injury. However, activated microglia have now been identified as homeostatic keepers in the brain. These microglia are also involved in the resolution of the neuroinflammatory response and neuroprotection.

Conversely, proinflammatory cytokines secreted by activated microglia and reactive species released by damaged neurons can trigger astrogliosis [4–6]. Reactive astrogliosis is a hallmark of the neuroprotective [7] or neurotoxic [8] reaction of astrocyte, depending on the context of damage. An astrogliosis further results in a characteristic structure called a glial scar. Glial scar formation prevents infiltration of peripheral inflammatory cells or molecules, whereas it can also be detrimental to neuronal recovery by constituting a physical and biochemical barrier that inhibits axonal regeneration [4, 9].

CatH (EC 3.4.22.16) is a lysosomal cysteine protease with a unique aminopeptidase activity, and its expression level is increased in activated immune cells [10, 11]. Recently, we have reported that CatH deficiency impaired toll-like receptor 3 (TLR3)-mediated activation of interferon regulatory factor 3 (IRF3) and consequent secretion of interferon-β (IFN-β) from dendritic cells [11]. Furthermore, there is increasing evidence that IFN-β secreted from microglia/macrophages has neuroprotective effects.

These observations prompted us to further investigate the precise roles of CatH in HI-induced neuropathological changes with a special focus on microglia by using genetic inhibition approaches. In this study, CatH deficiency revealed a novel mechanism underlying microglia-astrocyte communication that is crucial for brain damage after HI injury. In *CatH*^−/−^ mice, HI-induced hippocampal atrophy was significantly reduced. However, a significant neuronal death was still observed in the hippocampus. Therefore, it is conceivable that neuroprotection resulting from CatH deficiency is not responsible for the improvement of HI-induced hippocampal atrophy in *CatH*^−/−^ mice. Somewhat surprisingly, we found notable cell death of microglia/macrophages, strong reactive astrogliosis and reduced TLR3/IFN-β signaling molecules in the hippocampus of *CatH*^−/−^ mice following HI. Therefore, reduced TLR3/IFN-β signaling resulting from CatH deficiency is responsible for pathological changes observed in the hippocampus of *CatH*^−/−^ mice following HI.

## Methods

### Animals

Heterozygous mice of C57BL/6N background were kept under specific pathogen-free conditions at Kyushu University Faculty of Dental Sciences. Genotyping of *CatH*^−/−^ mice was performed according to the method described previously [12]. The wild-type (WT) mice were derived from the same breeding colony. All experimental procedures of this study were approved by the Animal Care and Use Committee of Kyushu University.

### HI injury model

A neonatal HI brain injury was essentially induced in WT and *CatH*^−/−^ mice at P7, using the methods of the Rice-Vannucci model, with minor modifications [13]. After inhalation anesthesia, the left common carotid artery was dissected and ligated with silk sutures (6/0). After the surgical procedure, the pups were allowed to recover for 1 h at 37 °C in an incubator. They were then placed in chambers maintained at 37 °C through which 8% humidified oxygen (balance, nitrogen) flowed for 45 min. After hypoxic exposure, the pups were returned to their dams and the plastic cages. The animals were allowed to recover for 72 h. The brains were processed for biochemical or morphological analyses. This procedure resulted in brain injury in the ipsilateral hemisphere, consisting of cerebral infarction that was mainly localized to the hippocampus. To minimize experimental variations, the same person conducted the surgery throughout this study. Furthermore, both WT and *CatH*^−/−^ mice were exposed to the hypoxic condition simultaneously by placing them in the same chamber.

### Hematoxylin-eosin staining

The histological changes in WT and *CatH*^−/−^ mice were performed using hematoxylin-eosin (H&E). Briefly, mouse brain sections (20 μm) were immersed in Hematoxylin (Sigma) solution for 5 min, rinsed vigorously with distilled water for 15 min, and immersed in Eosin Y solution for 30 s. The sections were then dehydrated by immersing in gradient ethanol (70%, 80%, 90%, 95%, and 100%) for 5 min and Xylene for 30 s. The sections were mounted in microscopy Entellan® new (Millipore). Images were taken using a microscope (BX-41, Olympus).

### Immunofluorescent staining

The brains from WT and *CatH*^−/−^ mice (P7) were obtained 72 h after HI. The samples were cryoprotected in 30% sucrose and embedded in an optimal cutting temperature (OCT) compound (Sakura Fine Technical). Frozen sections (20 μm) of the samples for immunofluorescent staining were prepared as previously reported [14]. The sections were incubated with antibodies: goat anti-CatH (1:1000; Santa Cruz Biotechnology) with rabbit anti-ionized calcium-binding adapter protein 1 (Iba1, 1:10,000; Wako), rabbit anti-glial fibrillary acidic protein (GFAP, 1: 5000; Sigma-Aldrich), or mouse anti-NeuN (1: 5000; Millipore), rabbit anti-Iba1 with NeuroTrace Fluorescent Nissl Stains (1:200; Molecular Probes), rabbit anti-TLR3 (1:500, Santa Cruz) with rat anti-F4/80 (1:1000; Abcam), rabbit anti-phosphorylated signal transducers and activators of transcription 1 (p-STAT1, 1:500; Cell signaling), at 4 °C overnight. After washing with phosphate-buffered saline (PBS), the sections were incubated with donkey anti-goat Alexa 488 (1:1000; Jackson ImmunoResearch), donkey anti-rabbit Cy3 (1:1000; Jackson ImmunoResearch) and donkey anti-mouse Cy3 (1:1000; Jackson ImmunoResearch), donkey anti-rabbit Alexa 488 (1:1000; Jackson ImmunoResearch), and donkey anti-rat Cy3 (1:1000; Jackson ImmunoResearch) at 4 °C for 2 h. The sections were mounted in Vectashield anti-fading medium (Vector Laboratories). The cultured microglia were fixed with 4% paraformaldehyde and incubated with the goat anti-CatH with rabbit anti-Rab5 (1:1000; Abcam) overnight at 4 °C. After washing with PBS, the sections were incubated with donkey anti-goat Alexa 488 (1:1000; Jackson ImmunoResearch) and donkey anti-rabbit Cy3 (1:1000; Jackson ImmunoResearch), then incubated with Hoechst (1:200) and mounted in Vectashield anti-fading medium. Fluorescence images were taken using a confocal microscope (C2si, Nikon).

### Cell culture

Primary cultured microglia were prepared from mixed cell cultures of the cerebral cortex from 3-day-old Wistar rats according to the previously described methods [15]. The cells were maintained in minimum essential medium (MEM, Gibco) containing 10% fetal bovine serum, 2 mg/ml Glucose, 1% Penicillin-Streptomycin, at 37 °C, and 10% CO_2_. The MG6 cell was a c-myc-immortalized mouse microglial cell line (Riken Cell Bank) which was maintained in Dulbecco’s modified Eagle medium (DMEM) with 10% fetal bovine serum (Gibco) supplemented with 10 μg/ml insulin, 1% penicillin-streptomycin (Gibco), and 2 mg/ml Glucose (Sigma). Primary astrocytes were prepared from mixed cell cultures of the cerebral cortex from neonatal mice according to the previously described methods [15]. After 10–14 days in culture, floating cells, weakly attached cells, and non-astroglial cells on the mixed glial cell layer were eliminated by shaking the flask. The resultant cell layer was then harvested and transferred to 24-well plates with polyethylenimine (PEI)-coated glasses.

### Pulse-chase experiments

Detailed procedure for pulse-chase experiments are described in previous studies [15]. Briefly, primary cultures of rat microglia (10^6^–10^7^ cells/dish) were preincubated at 37 °C for 90 min in methionine-free MEM containing 10% fetal calf serum. The cells were pulse-labeled for 30 min with [^35^S] methionine (> 37TBq/mmol, Du Pont-New England Nuclear; 1 mCi/2 ml per dish), and chased in fresh DMEM containing the excess of unlabeled methionine and 10% fetal calf serum. At the indicated times of chase, the cells were separated from the medium. After centrifugation, radiolabeled CatH in the precleared lysates and media were immunoreacted by incubation first with the anti-CatH antibody. Immune complexes were isolated by mixing at 4 °C for 3 h with 50 μl of an 80% suspension of protein A-Sepharose beads (Pharmacia LKB Biotechnology Inc.) in PBS with gentle agitation. The beads were boiled for 3 min at 100 °C, and the supernatant was analyzed by SDS-polyacrylamide gel electrophoresis (PAGE). Radioactive bands were detected by fluorography using Amplify (Amersham) on Konica Medical X-ray film. Apparent molecular weights were determined using ^14^C-methylated standards (Amersham).

### CatH knockdown with small interfering RNAs

MG6 cells were seeded in 96-well and 6-well plates. Twelve hours after seeding, the cells were transiently transfected with control siRNA and CatH siRNA (Invitrogen) using RNAi transfection reagent (Invitrogen) according to the manufacturer’s protocol. The CatH knockdown efficiency was examined by Western blotting.

### Real-time polymerase chain reaction

The mRNAs isolated from non- and poly(I:C)-treated MG6 microglia in the absence or presence of CatH siRNA were subjected to a real-time quantitative real-time polymerase chain reaction (RT-PCR). The total RNA was extracted with the RNAiso Plus (Takarada, Japan) according to the manufacturer’s instructions. A total of 1000 ng of extracted RNA was reverse transcribed to cDNA using the QuantiTect Reverse Transcription Kit (QIAGEN, Japan). After an initial denaturation step at 95 °C for 5 min, temperature cycling was initiated. Each cycle consisted of denaturation at 95 °C for 5 s, annealing at 60 °C for 10 s, and elongation for 30 s. In total, 40 cycles were performed. The cDNA was amplified in duplicate using a Rotor-Gene SYBR Green RT-PCR Kit (QIAGEN, Japan) with a Corbett Rotor-Gene RG-3000A Real-Time PCR System. The data were evaluated using the RG-3000A software program (version Rotor-Gene 6.1.93, Corbett). The sequences of primer pairs were as follows: CatH: 5′-TACAACAAGGGCATCATGGA-3′ and 5′-TTCTTGACGAATGCAACAGC-3′; TLR3: 5′-CCTCCAACTGTCTACCAGTTCC-3′ and 5′- GCCTGGCTAAGTTAT TGTGC-3′; IFN-β: 5′-AGGGCGGACTTCAAGATC-3′ and 5′-CTCATTCCACCC AGTGCT-3′; and Actin: 5′-AGAGGGAAATCGTGCGTGAC-3′ and 5′-CAATAG TGATGACCTGGCCGT-3′. For data normalization, an endogenous control (actin) was assessed to control the cDNA input, and the relative units were calculated using a comparative Ct method. All of the real-time RT-PCR experiments were repeated three times, and the results are presented as the means of the ratios ± standard deviation of the mean (SD).

### Enzyme-linked immunosorbent assay

The concentration of IFN-β in the cell culture medium prepared from non-, control siRNA-, and CatH siRNA-treated MG6 microglia subjected to standard and oxygen-glucose deprivation (OGD) conditions was quantitatively measured by enzyme-linked immunosorbent assay (ELISA) according to the manufacturer’s instructions (PBL Interferon Source).

### OGD and re-oxygenation

To mimic HI conditions in vitro, MG6 cells were exposed to glucose deprivation and hypoxia for 6 h. In the OGD phase, the various media were changed to glucose-free Hank’s balanced salt solution. The cells were then placed in a hypoxia incubator (Model: MCO 18M; Sanyo Biomedical Electrical Co., Ltd., Tokyo, Japan), which contained a gas mixture composed of 1% O_2_, 5% CO_2_, and 92% N_2_. After OGD, the cultures were replaced with DMEM and subjected to re-oxygenation in normoxia incubator (20% O_2_, 5% CO_2_).

### Isolation of splenocytes

WT and *CatH*^−/−^ mice anesthetized and perfused transcardially with PBS, and then the spleens were cut into small pieces. After enzymatic digestion using the Neural Tissue Dissociation Kit (Papain), the cell suspensions were further mechanically dissociated using a gentle MACS Dissociator (Milteny Biotec). The red blood cells were removed by Red Blood Cell Lysis Solution (Miltenyi Biotec).

### Western blotting

The cells extracts were prepared by ultrasonication in RIPA buffer in the presence of a protease inhibitor cocktail (Sigma). Proteins in the fraction were separated into 7.5%, and 12% SDS-polyacrylamide gels. After transfer and blocking, the PVDF membranes were incubated at 4 °C overnight under gentle agitation with each primary antibody: goat anti-CatH (1:500), rabbit anti-toll-like receptor 3 (TLR3, 1:500, Santa Cruz), rabbit anti-phosphorylated interferon regulatory factor 3 (p-IRF3, 1:1000, Cell Signaling Technology), rabbit anti-IRF3 (1:1000, Cell Signaling Technology), and mouse anti-actin (1:5000; Abcam). After washing, the membranes were incubated with horseradish peroxidase (HRP)-labeled anti-goat (1:2000; GE Healthcare), anti-mouse (1:2000 R&D Systems) and anti-rabbit (1:2000; GE Healthcare) for 2 h at room temperature. Subsequently, the membrane-bound, HRP-labeled antibodies were detected using an enhanced chemiluminescence detection system (ECL kit; GE Healthcare) with an image analyzer (LAS-3000; Fuji Photo Film).

### Cell viability assay

MG6 cells were seeded in 96-well plates at 10^4^ cells/well in a 100 μl suspension. After various treatments, a cell viability assay was conducted using a Cell-Counting Kit (CCK-8, Dojindo). CCK-8 ( μl) was added to each well and incubated in a CO_2_ incubator for 1 h. The optical density was read at a wavelength of 450 nm with a microplate reader, and the cell viability was calculated using the following formula: Cell viability = optical density of treated group/indicated control group.

### Flow cytometry

The MG6 cells were seeded in 6-well plates at a density of 2.5 × 10^5^ cells/ml. After various treatments, the cells were harvested and stained with Annexin V FITC (BioVision) according to the manufacturer’s protocol. A total of 5000 events were analyzed using flow cytometry (BD FACSVerse). Propidium Iodide (BioVision) was used to indicate the dead cells from flow cytometric analysis. The FACS data were analyzed by FlowJo software (Tree star).

### Scratch wound assay

The scratch wound assay is a common in vitro method used to measure cell migration/proliferation [16]. The capacity of the astrocytes to induce repopulation of the wound was examined by scratching confluent astrocyte monolayers. The wound was induced by dragging a sterile pipette tip (200 μl) across the surface of astrocyte monolayers. The detached cells and debris were removed immediately by washing with PBS. The cells were maintained for an additional 72 h in the presence or absence of 100 U/ml human recombinant IFN-β (PBL Assay Science). Bright-field images at the region of interest were taken at 0, 24, 48, and 72 h after incubation under an inverted microscope (Ti-S, Nikon). The cells were then fixed and stained for GFAP to further examine the effects of IFN-β on the migration/proliferation of astrocytes.

### Statistical analysis

The data are represented as mean ± SD. Statistical analyses were performed using a one-way ANOVA with a post hoc Tukey’s test and Student’s *t* test using the GraphPad Prism software package. A value of *p* < 0.05 was considered to indicate statistical significance (GraphPad Software).

## Results

### Reduction in HI-induced brain atrophy in the hippocampus resulting from CatH deficiency

**Fig. 1:**
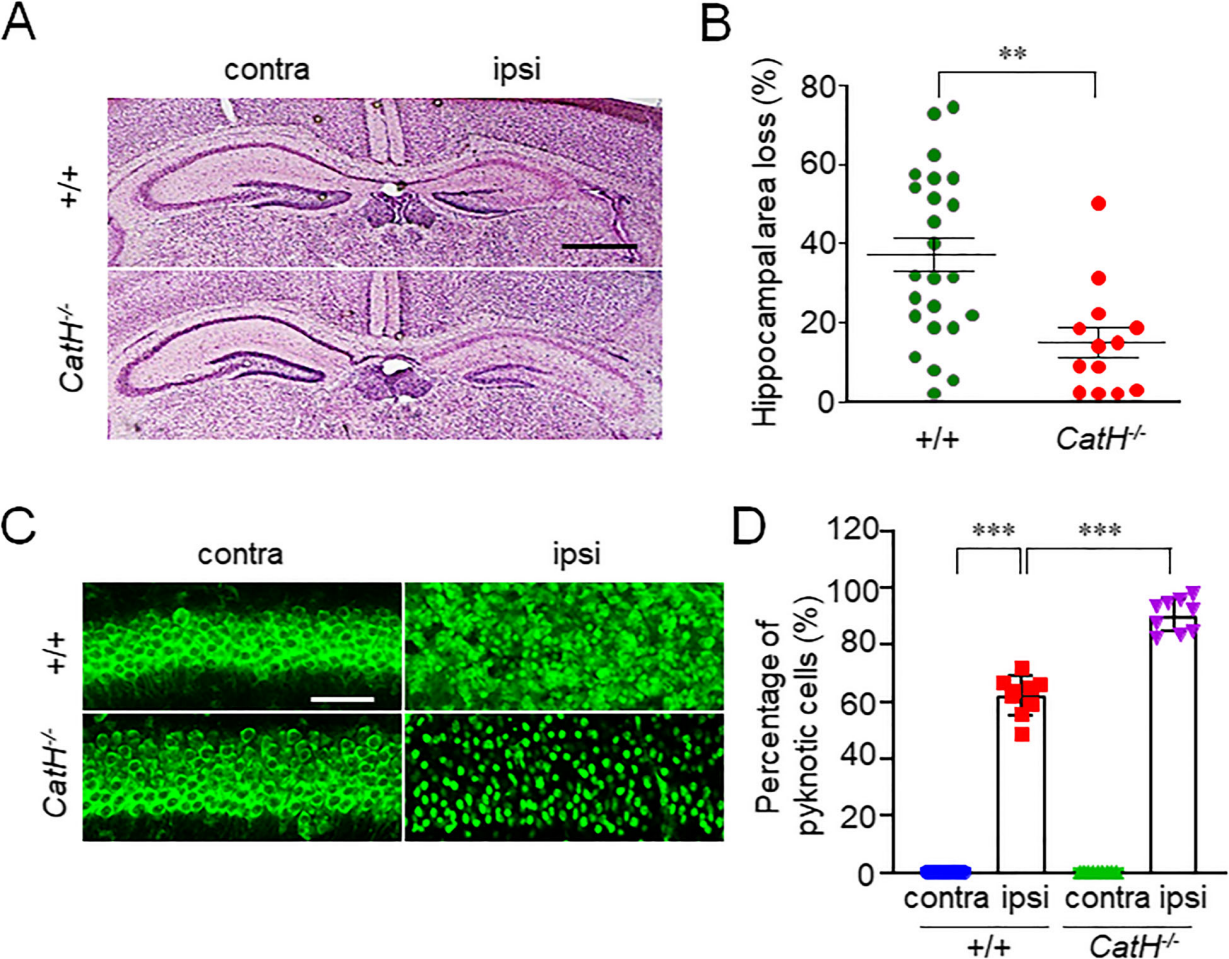
CatH deficiency prevents neuronal damage in hippocampus of neonatal mice 72 h after HI injury. **A** The histological changes in the hippocampus of WT and *CatH*^−/−^ mice72 h after HI injury indicated with HE staining. Scale bar, 400 μm. **B** The quantification of the ipsi hippocampal area loss of WT (*n* = 25) and *CatH*^−/−^ (*n* = 13) mice 72 h after HI injury. The asterisks indicate a statistically significant difference from the WT ipsi value (***p < 0.001, Student’s *t* tests). **C** Fluorescent CLSM images of the hippocampal region of WT and *CatH*^−/−^ mice 72 h after HI injury. Scale bar, 50 μm. **D** The quantification of the ratio of pyknotic neuronal cells in the hippocampal CA1 region of WT and *CatH*^−/−^ mice 72 h after HI injury. The columns and bars represent the mean ± SD (*n* = 6). The asterisks indicate a statistically significant difference from the indicated contra value (***p < 0.001, one-way ANOVA test). *Contra* contralateral, *ipsi* ipsilateral

To investigate the role of CatH in HI-induced neuronal damage, the pyramidal regions of the hippocampus ipsilateral to the ligated side of both the neonatal WT and *CatH*^−/−^ mice after HI were compared. The damaged area (%), 72 h after HI, was examined by calculating the ratio of shrunken area to the total area of the pyramidal regions of the hippocampus of the neonatal WT and *CatH*^−/−^ mice using H&E staining. Considerable damage, ranging from moderately severe to complete loss of pyramidal cell layer was observed in neonatal WT mice (Fig. 1A, B), which were consistent with the previously reported results [13, 17]. In contrast, the variation of the hippocampal damage in the neonatal *CatH*^−/−^ mice was minimal, and the mean ratio of the damaged area was significantly lower than in the neonatal WT mice (Fig. 1A, B).

Interestingly, even CatH deficiency prevented the shrinkage of the pyramidal regions of the hippocampus of neonatal mice, and the cell density was markedly lower than that in the contralateral side in both WT and *CatH*^−/−^ mice after HI (Fig. 1A). The results were further confirmed using the fluorescent Nissl stain. An increase in the number of pyknotic neurons was also observed in the pyramidal regions of the hippocampal of both WT and *CatH*^−/−^ mice after HI (Fig. 1C), while pyknotic neurons in *CatH*^−/−^ mice exhibited relatively well-defined cell margins and significantly higher cell number compared with WT mice after HI (Fig.1 C, D).

### Distribution and processing of CatH in the hippocampus after HI

**Fig. 2:**
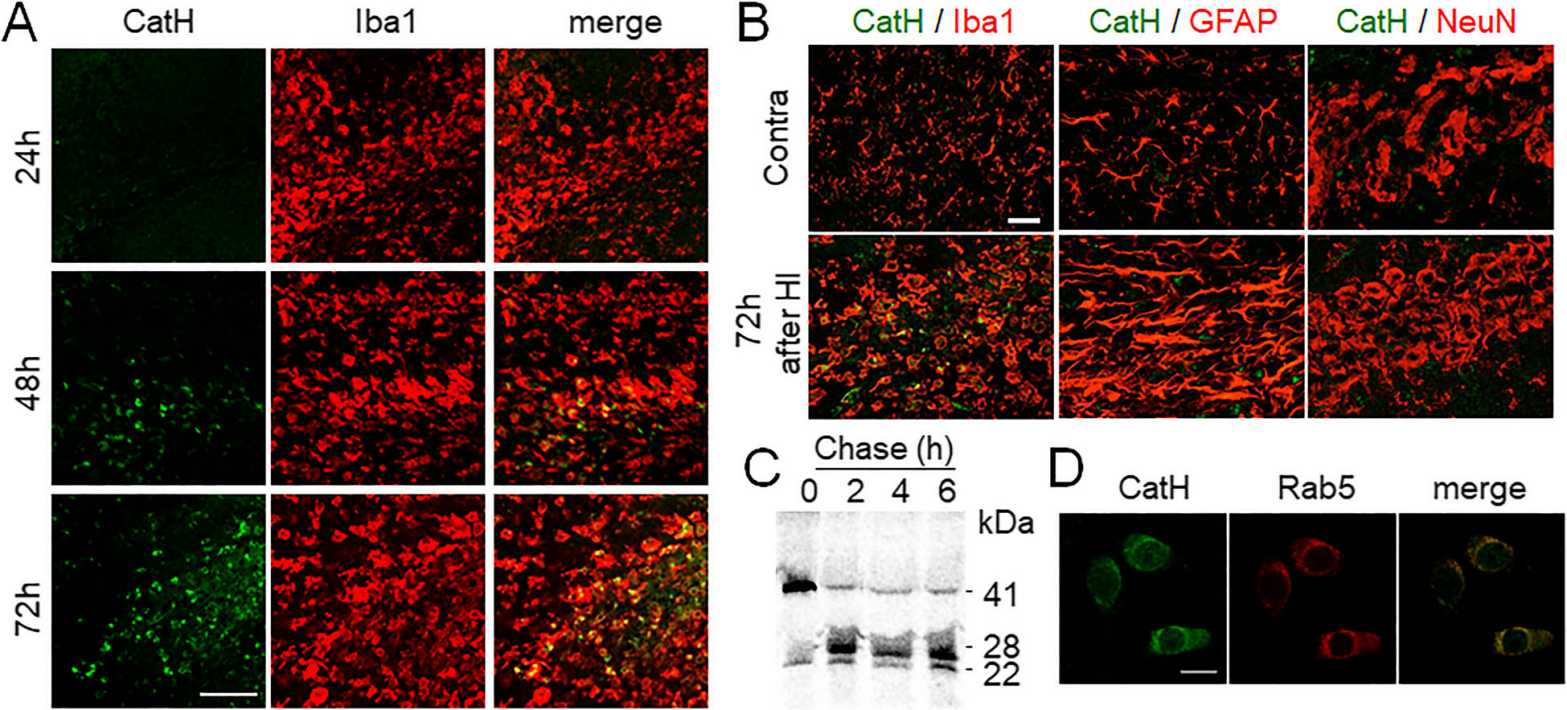
Distribution and processing of CatH in microglia. **A**, **B** Immunofluorescent CLSM images of CatH (green) with Iba1, GFAP, and NeuN (red) in the ipsi hippocampus of WT mice 72 h after HI injury. Scale bar, 50 μm. *Contra* contralateral, *ipsi* ipsilateral. **C** Pulse-chase analysis of CatH in primary cultured microglia. The cells were labeled with [^35^S] methionine for 30 min and chased for the indicated time. At indicated time, cell lysate was prepared and used for immunoprecipitation of CatH. The immunoprecipitates were analyzed by SDS-PAGE and fluorography. **D** Immunofluorescent CLSM images of CatH (green) and Rab5 (red) in microglia. Scale bar, 10 μm

Next, the expression and localization of CatH after HI were examined. The immunoreactivity for CatH in the pyramidal regions of the ipsilateral hippocampus was markedly increased from 48 h after HI (Fig. 2A). At 72 h after HI, intense CatH immunoreactivity was observed in Iba1^+^ cells (72 h after HI), but not in astrocytes and neurons (Fig. 2B). Iba1^+^ cells in the hippocampus consisted of resident microglia and infiltrating blood-derived macrophages even at 24 h after HI [18]. To further clarify the biosynthesis and processing of CatH in microglia, the primary cultures of microglia were labeled with [^35^S] methionine for 30 min and then chased for up to 6 h. Labeled polypeptides present in the cells and secreted in the medium were immunoprecipitated and analyzed by SDS-PAGE under reducing conditions followed by fluorography. CatH was initially observed as a polypeptide with an apparent molecular mass of 41 kDa (Fig. 2C), which corresponded well with pro-CatH. After a chase for 2 h, most of the polypeptide was converted to a 28-kDa polypeptide corresponding to the single-chain form. After a chase for 6 h, the 28-kDa polypeptide was slightly processed to a 22-kDa polypeptide corresponding to the heavy chain form. These results indicate that the initially synthesized pro-CatH with a molecular mass of 41 kDa rapidly underwent complete proteolytic processing yielding the mature form (28 kDa), which corresponded well with that in primary cultured rat hepatocytes [19]. However, the further processing of the single chain form to the double-chain form is relatively slow in microglial cells. Notably, a small amount (< 10%) of the precursor form of CatH was secreted as a polypeptide (41 kDa) and accumulated in the medium during a 2-h chase period. Microglia displayed punctate fluorescence of CatH immunoreactivity over the whole cytoplasm colocalized with Rab5, indicating the early endo-lysosomal localization of CatH (Fig. 2D).

### CatH deficiency prevents the increased expression of TLR3 by microglia/macrophages in the ipsilateral hippocampus after HI

**Fig. 3:**
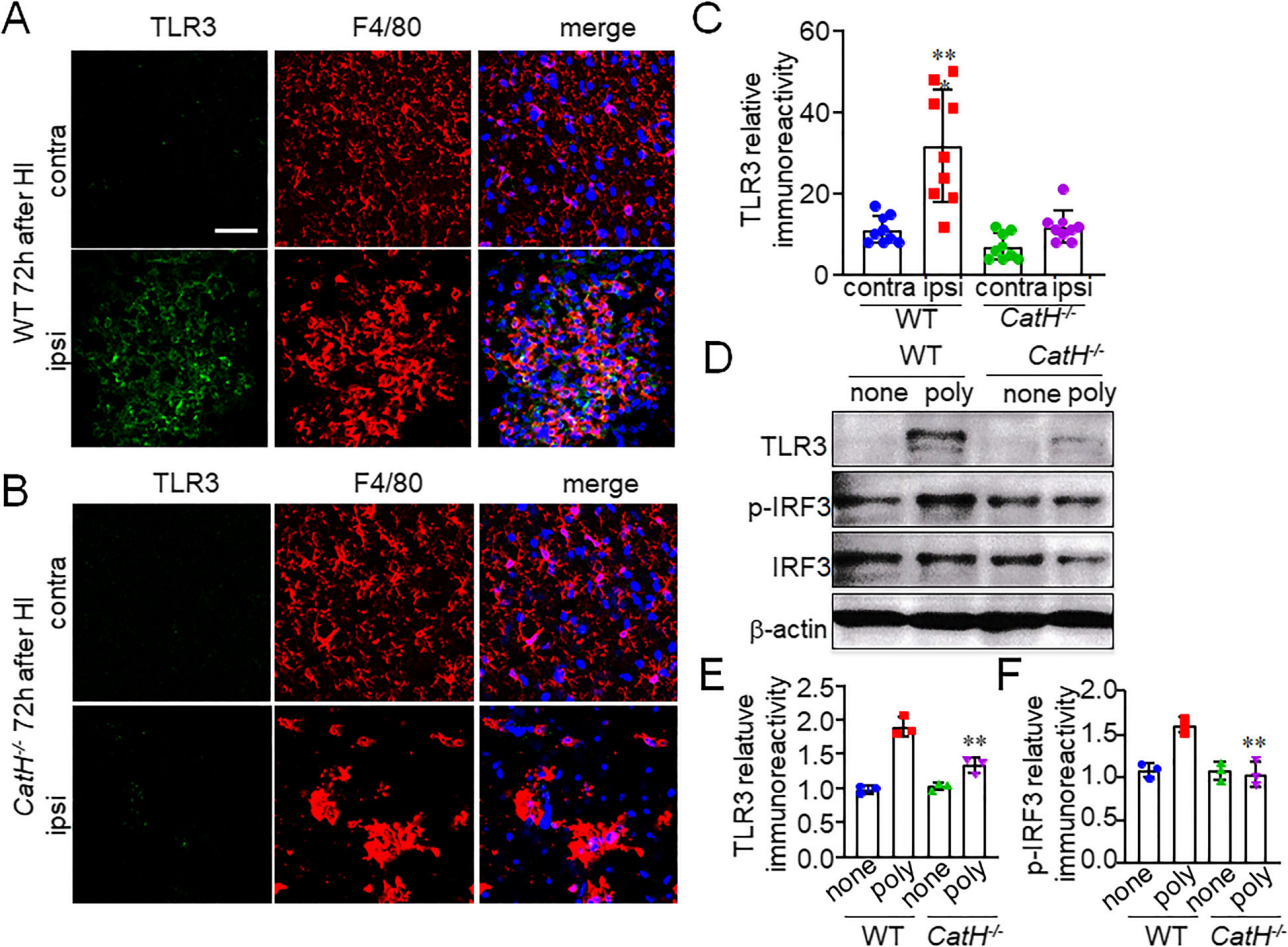
CatH deficiency prevents the increased expression of TLR3 in microglia/macrophages after HI. **A**, **B** CLSM images of TLR3 (green), F4/80 (red), and their merged images with overlay of Hoechst staining (blue) in the cortex of WT (**A**) and *CatH*^−/−^ (**B**) mice 72 h after HI injury. *Contra* contralateral, *ipsi* ipsilateral. Scale bar, 50 μm. **C** The mean relative immunoreactivity for TLR3 in the pyramidal regions of the hippocampus of WT and *CatH*^−/−^ mice. The columns and bars represent the mean ± SD (*n* = 9). The asterisks indicate a statistically significant difference from contra (**p < 0.01, one-way ANOVA test). **D** immunoblots show the full-length TLR3, p-IRF3, and IRF3 expression in splenocytes from WT and *CatH*^−/−^ mice 48 h after treatment with 10 μg/ml poly(I:C). **E**, **F** The quantitative analyses of TLR3 (**E**) and p-IRF3 (**F**) in the immunoblots in (**D**). The columns and bars represent the mean ± SD (*n* = 3). The asterisks indicate a statistically significant difference from WT poly group value (**p < 0.01, one-way ANOVA test)

TLR3 is known to be a substrate of CatH, and the N-terminal cleavage of TLR3 by CatH plays an important role in TLR3 maturation [20]. Therefore, we proposed that CatH may also involve in the activation of TLR3 in microglia/macrophages after HI. The TLR3 expression level was determined by immunofluorescent staining and immunoblot analyses with the use of rabbit antibody against TLR3 that recognizes the epitope corresponding to amino acids 26–325 mapping within an N-terminal domain. The relative immunoreactivity for TLR3 in the pyramidal regions of the hippocampus of WT mice was significantly increased in comparison to that in the contralateral side at 72 h after HI (Fig. 3A, C). Most of the TLR3 were expressed in F4/80^+^ cells (Fig. 3A). In contrast, there were no significant increase in the immunoreactivity for TLR3 in the pyramidal regions of the hippocampus of *CatH*^−/−^ mice at 72 h after HI (Fig. 3B, C). It was also noted that the number of F4/80^+^ cells with amoeboid morphology was markedly decreased in the pyramidal regions of the hippocampus of *CatH*^−/−^ mice (Fig. 3B).

Next, we tried to compare the protein levels of TLR3 and p-IRF3 in microglia acutely isolated from the hippocampus of WT and *CatH*^−/−^ mice. However, it was very hard to detect the protein bands of TLR3 and p-IRF3 even after treatment with poly(I:C) in microglia isolated from the hippocampus, probably because of localized expression of TLR3 in microglia of the hippocampus. We then decided to use splenocytes isolated from WT and *CatH*^−/−^ mice, because splenic macrophages/dendritic cells express TLR3. As shown in Fig. 3D, only faint bands corresponding to full-length TLR3 were observed in the soluble fractions of both WT and *CatH*^−/−^ splenocytes. After treatment with poly(I:C), the mean protein level of full-length TLR3 was significantly increased in WT splenocytes, but not in *CatH*^−/−^ splenocytes (Fig. 3D, E). Conversely, p-IRF3 was constitutively expressed in both WT and *CatH*^−/−^ splenocytes. Poly(I:C) significantly increased the mean protein level of p-IRF3 in WT splenocytes, but not in *CatH*^−/−^ splennocytes (Fig. 3D, F). These results suggest that CatH is critical in the expression and activation of TLR3/IRF3 signaling, which is necessary for IFN-β production in TLR3-expressing cells including microglia/macrophages.

### CatH deficiency prevents the increased expression of p-STAT1 by microglia/macrophages in the ipsilateral hippocampus after HI

**Fig. 4:**
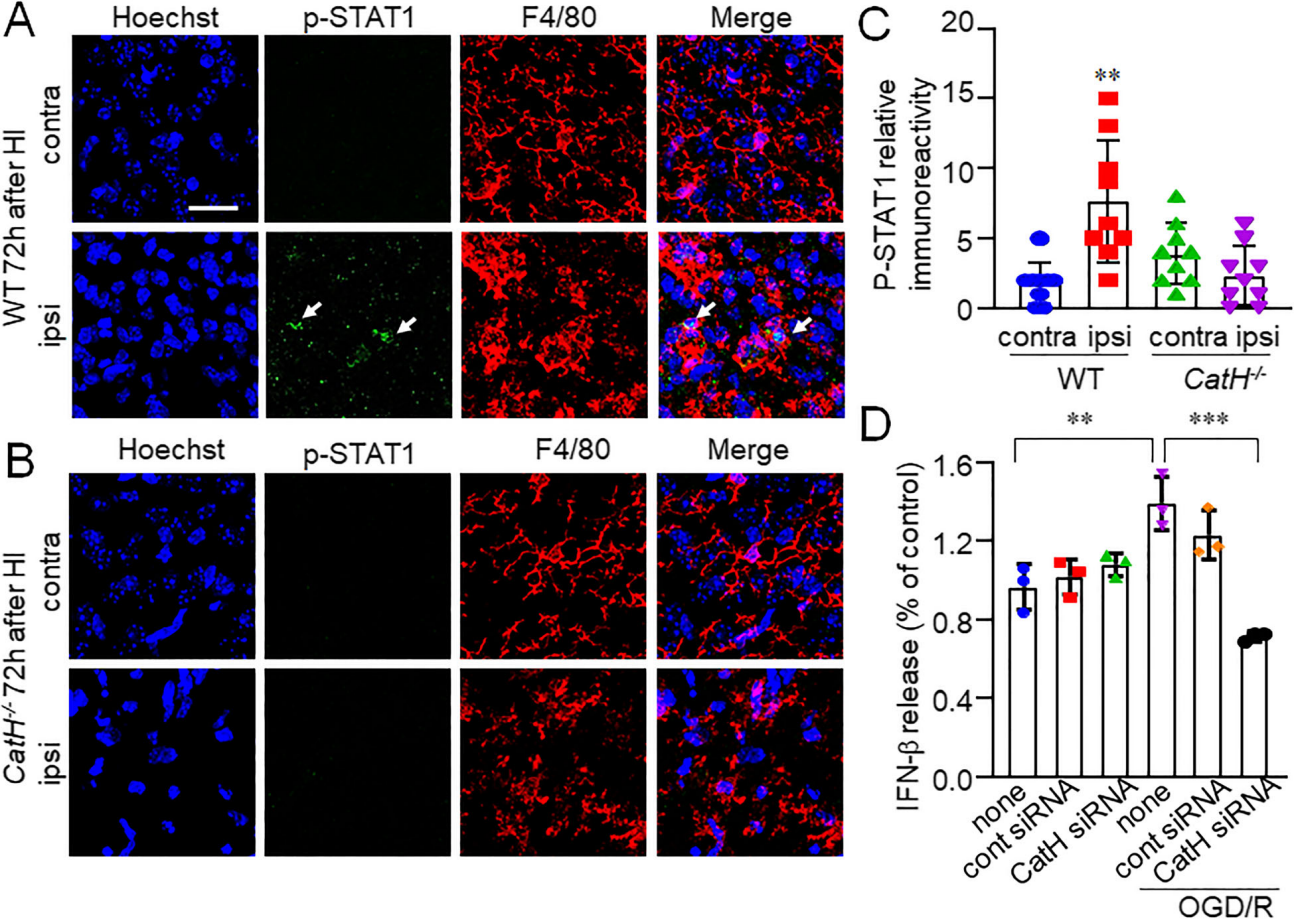
CatH deficiency prevents the increased expression of p-STAT1 in microglia/macrophages. **A**, **B** CLSM images of Hoechst (blue), p-STAT1 (green, arrows), F4/80 (red), and their merged images in the cortex of WT (**A**) and *CatH*^−/−^ (**B**) mice 72 h after HI injury. *Contra* contralateral, *ipsi* ipsilateral. Scale bar, 50 μm. **C** The mean relative immunoreactivity for p-STAT1 in the pyramidal regions of the hippocampus of WT and *CatH*^−/−^ mice. The columns and bars represent the mean ± SD (*n* = 9). The asterisks indicate a statistically significant difference from contra (**p < 0.01, one-way ANOVA test). **D** The amount of released IFN-β from the MG6 microglia after OGD/R in the presence of cont or CatH siRNA. The columns and bars represent the mean ± SD (*n* = 3). The asterisks indicate a statistically significant difference from indicated group (**p < 0.01 and ***p < 0.001, one-way ANOVA test)

We further detected a significant increase in immunoreactivity for p-STAT1 by use of rabbit monoclonal antibody against STAT-1 phosphorylated at Ser727 in activated microglia/macrophages of the ipsilateral hippocampus of WT mice after HI (Fig. 4A, C). In contrast, there was no significant increase in the immunoreactivity for p-STAT1in *CatH*^−/−^ mice after HI (Fig. 4B, C). We next examined effects of decreased expression of CatH on the secretion of IFN-β from MG6 microglia subjected to ODG. The mean amount of IFN-β secreted from MG6 microglia was significantly increased by OGD (Fig. 4D). CatH siRNA significantly inhibited the OGD-induced secretion of IFN-β from MG6 microglia (Fig. 4D).

These results suggest that microglia/macrophages in the ipsilateral hippocampus can secrete IFN-β to stimulate themselves in an autocrine manner, because p-STAT1 is a functional footprint of IFN-β. Furthermore, CatH is essential for the production of functional TLR3 and the subsequent production of IFN-β.

### Loss of activated microglia/macrophages in ipsilateral cortex and hippocampus after HI injury by CatH deficiency

**Fig. 5:**
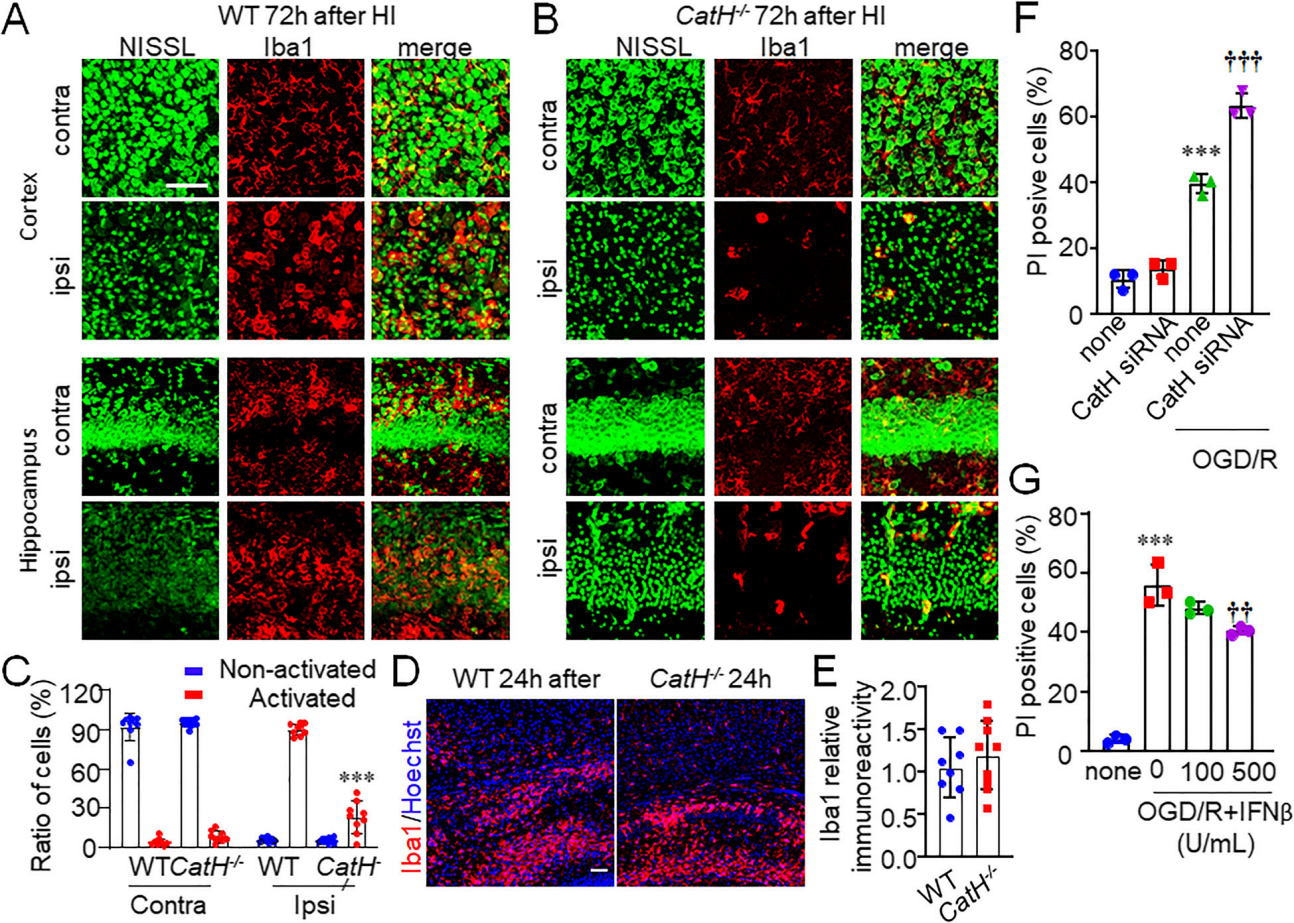
CatH deficiency induces cell death of microglia/macrophages in ipsilateral hippocampus after HI injury. **A**, **B** Immunofluorescent CLSM images of Nissl (green) and Iba1 (red) in contra and ipsi cortex and hippocampus of WT (A) and *CatH*^−/−^ (**B**) mice 72 h after HI injury. Scale bar, 100 μm. *Contra* contralateral, *ipsi* ipsilateral. **C** The quantitative analyses of cell number of activated and non-activated microglial cells in WT and *CatH*^−/−^ mice 72 h after HI injury. The columns and bars represent the mean ± SD (*n* = 9). The asterisks indicate a statistically significant difference from WT ipsi group (***p <0.001, one-way ANOVA test). **D** CLSM images of Hoechst (blue) and Iba1 (red) in the hippocampus of WT and *CatH*^−/−^ mice 24 h after HI injury. Scale bar, 100 μm. **E** The mean relative immunoreactivity for Iba1 in the pyramidal regions of the hippocampus of WT and *CatH*^−/−^ mice. The columns and bars represent the mean ± SD (*n* = 9). The asterisks indicate a statistically significant difference from the WT. **F** The quantitative analyses of percentage of apoptotic MG6 cells after subjected to OGD/R. The columns and bars represent the mean ± SD (*n* = 3). The asterisks indicate a statistically significant difference from control group value (***p < 0.001, one-way ANOVA test). The daggers indicate a statistically significant difference from OGD/R value (^†††^*p* < 0.001, one-way ANOVA test). **G** The quantitative analyses of percentage of apoptotic MG6 cells after subjected to OGD/R. The columns and bars represent the mean ± SD (*n* = 3). The asterisks indicate a statistically significant difference from control group value (***p < 0.01, one-way ANOVA test). The daggers indicate a statistically significant difference from OGD/R value (^**††**^*p* < 0.01, one-way ANOVA test)

The number of Iba1^+^ cells in the pyramidal regions of the hippocampus of *CatH*^−/−^ mice after HI was reduced compared to that in WT mice (Fig. 5B). Furthermore, most of Iba1^+^ microglia/macrophages phagocytosed the damaged neurons (Fig. 5A). These results indicated that microglia/macrophages in the *CatH*^−/−^ mice after HI may have low activation level or die after activation.

To address the mechanism for the reduced number of microglia/macrophages, the activation stage of microglia/macrophages was examined. No significant difference was observed in the mean cell number of Iba1^+^ microglia in the ipsilateral hippocampal CA1 subfield of WT and *CatH*^−/−^ mice at 24 h after HI injury (Fig. 5D, E). We further examined the mechanism by use of MG6 microglia. CatH siRNA significantly increased the oxygen-glucose deprivation and re-oxygenation (OGD/R)-induced apoptosis of MG6 microglia (Fig. 5F). To address the effect of IFN-β on the OGD/R-induced MG6 microglia, we applied human recombinant IFN-β to MG6 microglia. Pretreatment with 1000 U/ml IFN-β significantly inhibited the OGD/R-induced microglial cell death (Fig. 5G).

These observations supported the latter possibility. Furthermore, the results here further explained the data in Fig. 1C, D. Firstly, pyknotic neurons without well-defined cell margins may be phagocyted by activated microglia/macrophages and undergoing digestion in WT mice after HI, while the pyknotic neurons with well-defined cell margins may be consequence of escape from the phagocytosis by only small amount alive microglia/macrophages in the *CatH*^−/−^ mice after HI. Secondly, the less effective resolution of the pyknotic neurons may have resulted in maintaining of hippocampal structure in *CatH*^−/−^ mice after HI.

### Inhibition of astrocyte proliferation by IFN-β

**Fig. 6:**
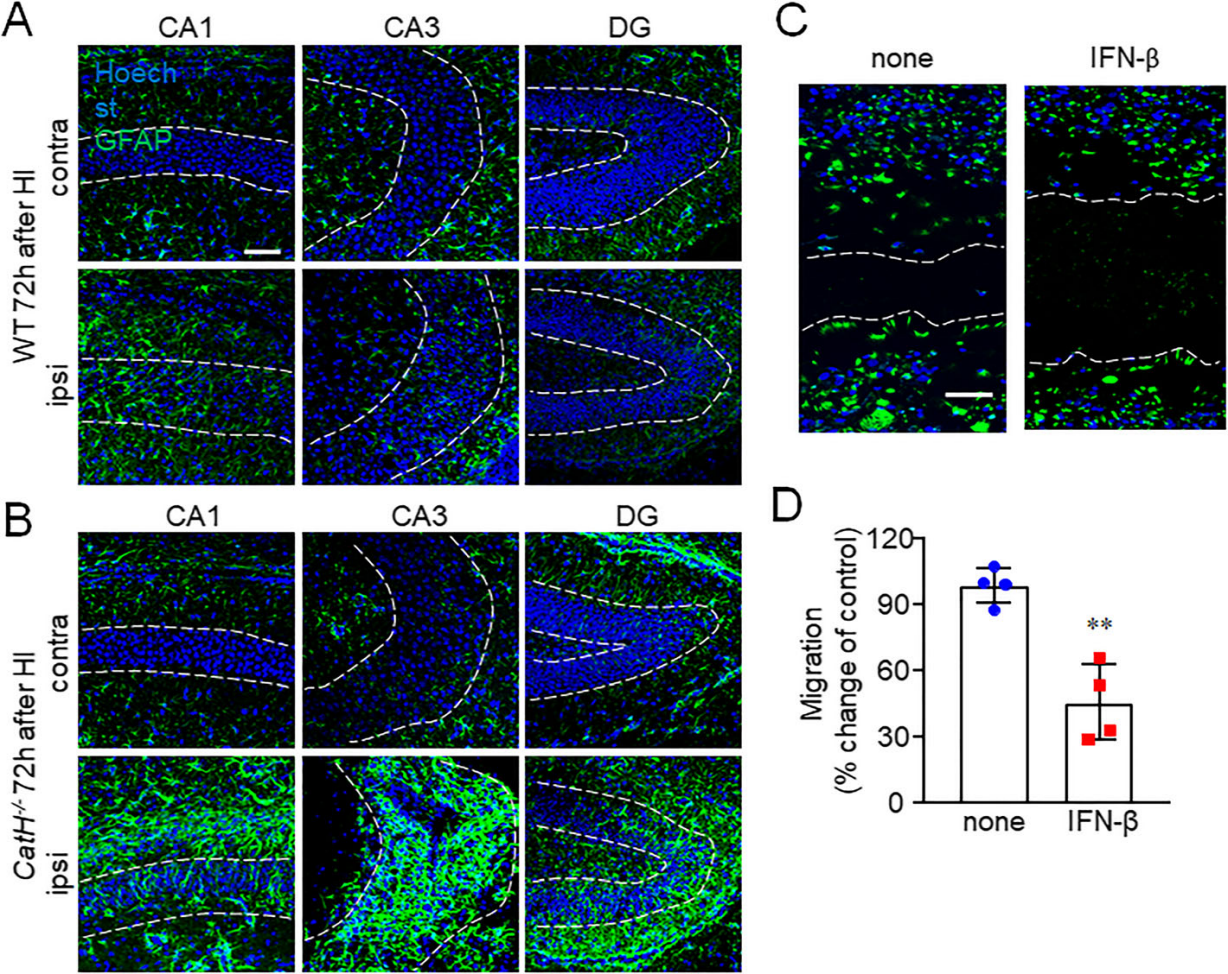
CatH deficiency enhanced the proliferation of astrocytes in the hippocampus after HI. **A**, **B** Immunofluorescent CLSM images of GFAP (green) and Hoechst (blue) in the hippocampal CA1, CA3, and dentate gyrus (DG) regions of WT (**A**) and *CatH*^−/−^ (**B**) mice at 72 h after HI injury. Scale bar, 100 μm. **C** CLSM images of the astrocyte monolayers immune-stained by anti-GFAP antibody (green) with nuclear staining by Hoechst (blue) in the astrocyte monolayers 72 h after the scratch in the absence and presence of IFN-β. Dashed lines highlight the area that was damaged by the scratch. Scale bar, 50 μm. **D** Quantification of the migration of cultured astrocytes shown in (**C**). The gap width from three separate experiments was quantitatively evaluated using ImageJ software. The asterisks indicate a statistically significant difference from control group value (**p < 0.05, unpaired *t* test)

The hippocampus in *CatH*^−/−^ mice was found to have a small number of activated microglia and pyknotic neurons without atrophy after HI, which promoted us to examine the possibility of other cell types that supported the structure of the hippocampus in *CatH*^−/−^ mice after HI. Histological estimates indicate that that there are at least five times more astrocytes than the central nervous system (CNS) neurons and that astrocytes occupy 50% of the total CNS volume. The cell density of the astrocytes was then examined by staining. Reactive astrocytes were only occasionally observed, and a glial scar was not very evident in the hippocampus of WT mice. Somewhat surprisingly, a large glial scar was formed by the accumulation of astrocytes in the hippocampal CA1, CA3, and dentate gyrus (DG) regions of *CatH*^−/−^ mice (Fig. 6A, B). Furthermore, the effects of IFN-β on the proliferation of astrocytes were examined by the scratch wound assay. Primary astrocytes were cultured and responses to the mechanical injury induced by the scratch wound were observed in a time-dependent manner. Pretreatment with IFN-β significantly reduced the accumulation of the reactive astrocytes in the scratch (Fig. 6C, D), thereby supporting the role of IFN-β in the hippocampal structure maintenance by astrocytes after HI injury.

## Discussion

In this study, the immunoreactivity for TLR3 was negligible in the hippocampus of either WT or *CatH*^−/−^ mice. After being subjected to HI, the TLR3 immunoreactivity was markedly increased in the ipsilateral hippocampus of WT mice, but not *CatH*^−/−^ mice. Similar results were also observed in the splenocytes, where only a faint protein band corresponding to TLR3 in both WT and CatH^−/−^ mice was detected. After stimulation with poly(I:C), the mean protein levels of TLR3 and p-IRF3 were significantly increased in the splenocytes prepared from WT mice, but not from *CatH*^−/−^ mice. These observations may be explained by the requirement for CatH in the maturation and stability of TLR3.

The full-length TLR3 is continuously exported to the Golgi apparatus and then rapidly transported to the endolysosomes where it undergoes a single cleavage by cathepsins [21]. Cathepsins process TLR3 within Loop1 of the leucine-rich repeat 12. When proteolytic cleavage is inhibited by either a cathepsin inhibitor or deletion of Loop1, TLR3 can still be activated by poly(I:C) in many types of cell lines that express transiently transfected TLR3. Although proteolytic processing is not required for TLR3 signaling [22], unprocessed TLR3 is degraded more rapidly than processed TLR3 fragments, suggesting that the CatH deficiency shifted TLR3 localization to endosomes and lysosomes recycling. In the present study, the mean protein level of TLR3 was very low, because the anti-TLR3 antibody used in this study detects only full-length TLR3. Furthermore, the mean protein level of full-length TLR3 was not increased in splenic macrophages prepared from *CatH*^−/−^ mice even after stimulation with poly(I:C) in this study, probably because the unprocessed TLR3 was degraded more rapidly in the endolysosomes [22], suggesting that the CatH deficiency shifted TLR3 localization to endosomes and lysosomes recycling. Therefore, we can infer that the TLR3 protein level in the neonatal mouse brain is tightly regulated, because TLR3 signaling inhibits the proliferation of neural stem/progenitor cells and the outgrowth of developing neurons [23]. Upon stimulation, the biosynthesis and maturation of TLR3 are promoted in a CatH-dependent manner. Besides viral double-stranded RNA, TLR3 can recognize self-nucleic acids [24] or endogenous protein agonists such as stathmin [25].

We found that WT mice displayed severe atrophy in association with neuronal death in the hippocampus following HI. In contrast, *CatH*^−/−^ mice did not exhibit marked atrophy in the hippocampus following HI, suggesting the inhibition of neuronal death. However, detailed histological analyses revealed a marked cell death of neurons and microglia/macrophages and excessive astrogliosis in the hippocampus. The reduction of TLR3/IFN-β signaling might be responsible for the HI-induced pathological changes observed in *CatH*^−/−^ mice. There is increasing evidence that IFN-β secreted from microglia has neuroprotective effects [26–28]. IFN-β also suppresses the production of nitric oxide (NO) and TNF-β [29], which are produced by activated microglia and are critical for the activation-induced cell death of microglia [30]. Therefore, it is likely that the reduced level of TLR3/IFN-β signaling resulting from CatH deficiency may induce further neuronal death and activation-induced cell death of microglia/macrophages in the hippocampus following HI. However, the role of TLR3/IFN-β signaling in the progression of ischemic brain injury remains the subject of debate. The activation of TLR3 markedly increased the neonatal brain’s vulnerability to HI injury in a toll/interleukin receptor domain-containing adaptor-inducing IFN-β (TRIF)-dependent manner [31]. However, stimulation of the TRIF pathway may be neuroprotective because it reprograms the cerebral response to stroke [32].

HI injury includes astrogliosis, neuroinflammation, and tissue repair and remodeling, which further contributes to brain cell loss and cerebral atrophy. Pro-inflammatory cytokines and reactive species released by damaged neurons under HI conditions can trigger astrogliosis [33]. Although astrogliosis is considered to exert beneficial effects, excessive reactive astrogliosis can impair neuronal recovery by forming the glial scar that can be detrimental to neuronal recovery by constituting a physical and biochemical barrier that inhibits axonal regeneration. In this study, we found marked astrogliosis without severe atrophy in the hippocampus of *CatH*^−/−^ mice following HI. Recombinant IFN-β significantly suppressed the migration/proliferation of primary cultured astrocytes. After migration into the injury sites, reactive astrocytes initiated glial scar formation [34]. Therefore, a reduced level of IFN-β secreted from activated microglia/macrophages following HI is also responsible for the large glial scar formation associated with the accumulation of reactive astrocytes in the hippocampus of *CatH*^−/−^ mice, leading to the reduction of cerebral atrophy. Our study’s results demonstrated that the attenuation of large glial scar formation by TLR3/IFN-β signaling is consistent with previous studies [35, 36]. To address the contribution of cerebral atrophy and glial scar in the brain recovery after HI injury, we will compare the restoration of brain functions and behavioral performances of WT and *CatH*^−/−^ mice in future studies.

Finally, it is interesting to discuss the differential function of CatB, another typical cysteine protease, and CatH in the HI model. CatB can activate TLR4/nuclear factor-κB (NFκB) signaling pathway through autolysosomal degradation of inhibitor of κBα (IκBα) in the HI macrophages/microglia [10, 13, 37]. Although we speculated that CatH might have the same functions as CatB in the HI model, the present study has revealed their differential pathological roles. Our previous [11] and the present studies suggest that CatH is necessary for the activation of TLR3/IRF3 signaling and consequent secretion of IFN-β through proteolytic maturation and stabilization of TLR3. It is noted that TLR4 and TLR3 are differentially involved in the cerebral HI damage. TLR4 activation exacerbates the cerebral HI damage through induction of neuroinflammation [38], whereas TLR3 activation induces protection against it [39]. Therefore, besides digestive enzymes, CatH and CatB are inflammatory and immune response-associated enzymes in microglia/macrophages through differential TLR signaling pathways in the HI model.

## Conclusion

We found that CatH plays a critical role in the proteolytic maturation and stabilization of TLR3, which is necessary for the TLR3/IFN-β signaling. Therefore, it may be concluded that impaired TLR3/IFN-β signaling resulting from CatH deficiency induces microglial cell death after activation and astrogliosis/glial scar formation in the hippocampus following HI injury, leading to suppression of hippocampal atrophy.

## Data Availability

The data used in this study are available from the corresponding authors up on reasonable request.

## Abbreviations

CatB: Cathepsin B
CatH: Cathepsin H
*CatH*^−/−^: Cathepsin H-deficient
CLMS: Confocal laser scanning microscope
CNS: Central nervous system
DG: Dentate gyrus
DMEM: Dulbecco’s modified Eagle medium
ELISA: Enzyme-linked immunosorbent assay
GFAP: Glial fibrillary acidic protein
HE: Hematoxylin-eosin
HI: Hypoxia-ischemia
HRP: Horseradish peroxidase
Iba1: Ionized calcium binding adapter protein 1
IFN-β: Interferon-β
IRF3: Interferon regulatory factor 3
IκBα: Inhibitor of κBα
MEM: Minimum essential medium
NFκB: Nuclear factor-κB
PAGE: Polyacrylamide gel electrophoresis
PBS: Phosphate-buffered-saline
PCR: Polymerase chain reaction
OGD/R: Oxygen-glucose deprivation and re-oxygenation
Poly(I:C): Polyinosine-polycytidylic acid
p-STAT1: Phosphorylated signal transducer and activators of transcription
SD: Standard deviation
TLR3: Toll-like receptor 3
WT: Wild-type

## References

[1] du Plessis AJ, Volpe JJ (2002). Perinatal brain injury in the preterm and term newborn. Curr Opin Neurol.

[2] Herrera MI, Mucci S, Barreto GE, Kolliker-Frers R, Capani F (2017). Neuroprotection in hypoxic-ischemic brain injury targeting glial cells. Curr Pharm Des.

[3] Lana D, Ugolini F, Giovannini MG (2020). An overview on the differential interplay among neurons-astrocytes-microglia in CA1 and CA3 hippocampus in hypoxia/ischemia. Front Cell Neurosci.

[4] Sofroniew MV (2009). Molecular dissection of reactive astrogliosis and glial scar formation. Trends Neurosci.

[5] Shinozaki Y, Shibata K, Yoshida K, Shigetomi E, Gachet C, Ikenaka K, Tanaka KF, Koizumi S (2017). Transformation of astrocytes to a neuroprotective phenotype by microglia via P2Y1 receptor downregulation. Cell Rep.

[6] Soheila KA, Rohini B (2012). Reactive astrogliosis after spinal cord 6. injury—beneficial and detrimental effects. Mol Neurobiol.

[7] Anderson MA, Burda JE, Ren Y, Ao Y, O'Shea TM, Kawaguchi R, Coppola G, Khakh BS, Deming TJ, Sofroniew MV (2016). Astrocyte scar formation aids central nervous system axon regeneration. Nature.

[8] Liddelow SA, Guttenplan KA, Clarke LE, Bennett FC, Bohlen CJ, Schirmer L, Bennett ML, Munch AE, Chung WS, Peterson TC (2017). Neurotoxic reactive astrocytes are induced by activated microglia. Nature.

[9] Silver J, Miller JH (2004). Regeneration beyond the glial scar. Nat Rev Neurosci.

[10] Nakanishi H (1868). Cathepsin regulation on microglial function. Biochim Biophys Acta Proteins Proteom.

[11] Okada R, Zhang X, Harada Y, Wu Z, Nakanishi H (2018). Cathepsin H deficiency in mice induces excess Th1 cell activation and early-onset of EAE though impairment of toll-like receptor 3 cascade. Inflamm Res.

[12] Buhling F, Kouadio M, Chwieralski CE, Kern U, Hohlfeld JM, Klemm N, Friedrichs N, Roth W, Deussing JM, Peters C, Reinheckel T (2011). Gene targeting of the cysteine peptidase cathepsin H impairs lung surfactant in mice. PLoS One.

[13] Ni J, Wu Z, Peterts C, Yamamoto K, Qing H, Nakanishi H (2015). The critical role of proteolytic relay through cathepsins B and E in the phenotypic change of microglia/macrophage. J Neurosci.

[14] Ni J, Wu Z, Stoka V, Meng J, Hayashi Y, Peters C, Qing H, Turk V, Nakanishi H (2019). Increased expression and altered subcellular distribution of cathepsin B in microglia induce cognitive impairment through oxidative stress and inflammatory response in mice. Aging Cell.

[15] Sastradipura DF, Nakanishi H, Tsukuba T, Nishishita K, Sakai H, Kato Y, Gotow T, Uchiyama Y, Yamamoto K (1998). Identification of cellular compartments involved in processing of cathepsin E in primary cultures of rat microglia. J Neurochem.

[16] Liang CC, Park AY, Guan JL (2007). In vitro scratch assay: a convenient and inexpensive method for analysis of cell migration in vitro. Nat Protoc.

[17] Koike M, Shibata M, Tadakoshi M, Gotoh K, Komatsu M, Waguri S, Kawahara N, Kuida K, Nagata S, Kominami E, Tanaka K, Uchiyama Y (2008). Inhibition of autophagy prevents hippocampal pyramidal neuron death after hypoxic-ischemic injury. Am J Pathol.

[18] Umekawa T, Osman AM, Han W, Ikeda T, Blomgren K (2015). Resident microglia, rather than blood-derived macrophages, contribute to the earlier and more pronounced inflammatory reaction in the immature compared with the adult hippocampus after hypoxia-ischemia. Glia.

[19] Tanaka Y, Tanaka R, Himeno M (2000). Lysosomal cysteine protease, cathepsin H, is targeted to lysosomes by the mannose 6-phosphate-independent system in rat hepatocytes. Biol Pharm Bull.

[20] Garcia-Cattaneo A, Gobert FX, Muller M, Toscano F, Flores M, Lescure A, Del Nery E, Benaroch P (2012). Cleavage of toll-like receptor 3 by cathepsins B and H is essential for signaling. Proc Natl Acad Sci U S A.

[21] Toscano F, Estornes Y, Virard F, Garcia-Cattaneo A, Pierrot A, Vanbervliet B, Bonnin M, Ciancanelli MJ, Zhang SY, Funami K, Seya T, Matsumoto M, Pin JJ, Casanova JL, Renno T, Lebecque S (2013). Cleaved/associated TLR3 represents the primary form of the signaling receptor. J Immunol.

[22] Qi R, Singh D, Kao CC (2012). Proteolytic processing regulates Toll-like receptor 3 stability and endosomal localization. J Biol Chem.

[23] Lathia JD, Okun E, Tang SC, Griffioen K, Cheng A, Mughal MR, Laryea G, Selvaraj PK, ffrench-Constant C, Magnus T (2008). Toll-like receptor 3 is a negative regulator of embryonic neural progenitor cell proliferation. J Neurosci.

[24] Bernard JJ, Cowing-Zitron C, Nakatsuji T, Muehleisen B, Muto J, Borkowski AW, Martinez L, Greidinger EL, Yu BD, Gallo RL (2012). Ultraviolet radiation damages self noncoding RNA and is detected by TLR3. Nat Med.

[25] Bsibsi M, Bajramovic JJ, Vogt MH, van Duijvenvoorden E, Baghat A, Persoon-Deen C, Tielen F, Verbeek R, Huitinga I, Ryffel B (2010). The microtubule regulator stathmin is an endogenous protein agonist for TLR3. J Immunol.

[26] Blank T, Prinz M (2017). Type I interferon pathway in CNS homeostasis and neurological disorders. Glia.

[27] Lobo-Silva D, Carriche GM, Castro AG, Roque S, Saraiva M (2017). Interferon-beta regulates the production of IL-10 by toll-like receptor-activated microglia. Glia.

[28] Scheu S, Ali S, Mann-Nuttel R, Richter L, Arolt V, Dannlowski U, et al. Interferon beta-mediated protective functions of microglia in central nervous system autoimmunity. Int J Mol Sci. 2019;20(1). 10.3390/ijms20010190. 10.3390/ijms20010190 PMC6337097 30621022

[29] Lubina-Dabrowska N, Stepien A, Sulkowski G, Dabrowska-Bouta B, Langfort J, Chalimoniuk M (2017). Effects of IFN-beta1a and IFN-beta1b treatment on the expression of cytokines, inducible NOS (NOS type II), and myelin proteins in animal model of multiple sclerosis. Arch Immunol Ther Exp (Warsz).

[30] Lee P, Lee J, Kim S, Lee MS, Yagita H, Kim SY, Kim H, Suk K (2001). NO as an autocrine mediator in the apoptosis of activated microglial cells: correlation between activation and apoptosis of microglial cells. Brain Res.

[31] Stridh L, Mottahedin A, Johansson ME, Valdez RC, Northington F, Wang X, Mallard C (2013). Toll-like receptor-3 activation increases the vulnerability of the neonatal brain to hypoxia-ischemia. J Neurosci.

[32] Marsh B, Stevens SL, Packard AE, Gopalan B, Hunter B, Leung PY, Harrington CA, Stenzel-Poore MP (2009). Systemic lipopolysaccharide protects the brain from ischemic injury by reprogramming the response of the brain to stroke: a critical role for IRF3. J Neurosci.

[33] Li B, Concepcion K, Meng X, Zhang L (2017). Brain-immune interactions in perinatal hypoxic-ischemic brain injury. Prog Neurobiol.

[34] Hsu JY, Bourguignon LY, Adams CM, Peyrollier K, Zhang H, Fandel T, Cun CL, Werb Z, Noble-Haeusslein LJ (2008). Matrix metalloproteinase-9 facilitates glial scar formation in the injured spinal cord. J Neurosci.

[35] Nishimura Y, Natsume A, Ito M, Hara M, Motomura K, Fukuyama R, Sumiyoshi N, Aoki I, Saga T, Lee HJ, Wakabayashi T, Kim SU (2013). Interferon-beta delivery via human neural stem cell abates glial scar formation in spinal cord injury. Cell Transplant.

[36] Li Y, Xu XL, Zhao D, Pan LN, Huang CW, Guo LJ, Lu Q, Wang J (2015). TLR3 ligand poly IC attenuates reactive astrogliosis and improves recovery of rats after focal cerebral ischemia. CNS Neurosci Ther.

[37] Nakanishi H (2020). Microglial cathepsin B as a key driver of inflammatory brain diseases and brain aging. Neural Regen Res.

[38] Zhang X, Ha T, Lu C, Lam F, Liu L, Schweitzer J, Kalbfleisch J, Kao RL, Williams DL, Li C (2015). Poly (I:C) therapy decreases cerebral ischaemia/reperfusion injury via TLR3-mediated prevention of Fas/FADD interaction. J Cell Mol Med.

[39] Takeuchi O, Akira S (2010). Pattern recognition receptors and inflammation. Cell.

